# TRPM8 Channel Activation Reduces the Spontaneous Contractions in Human Distal Colon

**DOI:** 10.3390/ijms21155403

**Published:** 2020-07-29

**Authors:** Antonella Amato, Simona Terzo, Laura Lentini, Pierenrico Marchesa, Flavia Mulè

**Affiliations:** 1 Department of Biological-Chemical- Pharmaceutical Science and Technology (STEBICEF), University of Palermo, Viale delle Scienze, Edificio 16, 90128 Palermo, Italy; simona.terzo01@unipa.it (S.T.); laura.lentini@unipa.it (L.L.); flavia.mule@unipa.it (F.M.); 2 Department of Biomedicine, Neurosciences and Advanced Diagnostics (BIND), University of Palermo, Via del Vespro 129, 90127 Palermo, Italy; 3 U.O. Oncology Hospital, A.R.N.A.S. Ospedali Civico Di Cristina Benfratelli, Palermo, Via Carmelo Lazzaro, 4, 90127 Palermo, Italy; pmarchesa1@gmail.com

**Keywords:** TRPM-8, 1-[Diisopropyl-phosphinoyl]-alkane (DIPA), human colon contractility, IBS

## Abstract

The transient receptor potential-melastatin 8 (TRPM8) is a non-selective Ca^2+^-permeable channel, activated by cold, membrane depolarization, and different cooling compounds. TRPM8 expression has been found in gut mucosal, submucosal, and muscular nerve endings. Although TRPM8 plays a role in pathological conditions, being involved in visceral pain and inflammation, the physiological functions in the digestive system remain unclear as yet. The aims of the present study were: (i) to verify the TRPM8 expression in human distal colon; (ii) to examine the effects of TRPM8 activation on colonic contractility; (iii) to characterize the mechanism of action. Reverse transcriptase-polymerase chain reaction (RT-PCR) and western blotting were used to analyze TRPM8 expression. The responses of human colon circular strips to different TRPM8 agonists [1-[Dialkyl-phosphinoyl]-alkane (DAPA) 2–5, 1-[Diisopropyl-phosphinoyl]-alkane (DIPA) 1–7, DIPA 1–8, DIPA 1–9, DIPA 1–10, and DIPA 1–12) were recorded using a vertical organ bath. The biomolecular analysis revealed gene and protein expression of TRPM8 in both mucosal and smooth muscle layers. All the agonists tested, except-DIPA 1–12, produced a concentration-dependent decrease in spontaneous contraction amplitude. The effect was significantly antagonized by 5-benzyloxytryptamine, a TRPM8 antagonist. The DIPA 1–8 agonist resulted in the most efficacious and potent activation among the tested molecules. The DIPA 1–8 effects were not affected by tetrodotoxin, a neural blocker, but they were significantly reduced by tetraethylammonium chloride, a non-selective blocker of K^+^ channels. Moreover, iberiotoxin, a blocker of the large-conductance Ca^2+^-dependent K^+^-channels, but not apamin, a blocker of small-conductance Ca^2+^-dependent K^+^ channels, significantly reduced the inhibitory DIPA 1–8 actions. The results of the present study demonstrated that TRPM8 receptors are also expressed in human distal colon in healthy conditions and that ligand-dependent TRPM8 activation is able to reduce the colonic spontaneous motility, probably by the opening of the large-conductance Ca^2+^-dependent K^+^-channels.

## 1. Introduction

Transient receptor potential (TRP) channels constitute a large family of non-selective cation channels involved in diverse cellular functions [1,2]. They display large diversity in activation mode, ion selectivity and, consequently, physiological functions. Most TRP channels are distributed in both the somatic and visceral sensory nervous systems, playing a crucial role in chemo-, thermo- and mechano-sensation [3,4]. The TRPM (transient receptor potential melastatin) represents the largest and most variable subfamily of the TRP channels, being constituted by eight members, from TRPM1 to TRPM8, and responding to various stimuli such as changes in the concentration of ions, small molecules, and lipids [2]. The TRPM channels promote Ca^2+^ entry into the cytosol resulting from directly Ca^2+^-permeable channels or indirectly from conductance changes of other channels.

TRPM8 is a member of the temperature-sensitive TRP channels. It is activated by mild cold temperatures, membrane depolarization, changes in extracellular osmolarity, cooling compounds such as menthol and icilin, and it shows a modest permeability to calcium [5,6]. TRPM8 is mainly expressed in sensory neurons innervating the skin, oral cavity [7], bladder [8], lungs [9], and prostate [10], and its activation seems to be involved in the relief of pain in neuropathic conditions or tissue inflammation [1]. TRPM8 is thought to have a role in the inhibition of visceral pain signals and reduction of inflammatory conditions in irritable bowel syndrome (IBS) as TRPM8 activation reduces colon inflammation both in animal models [4] and human subjects [11,12]. TRPM8 channels have been implicated also in oropharyngeal dysphagia [13] and chronic cough [14], while the downregulation of TRPM8 by angiotensin II may be involved in hypertension [15].

Although increasing evidence supports the important contribution of TRPM8 channels to the regulation of the inflammatory responses, making them potential targets in the treatment of IBS [1,16], the precise functional implications of TRPM8 in the gut remain unclear. Firstly, the intestinal TRPM8 expression has long been controversial, because positive [17] and negative [18] evidence was reported. TRPM8 transcript expression has been reported in the afferent neurons, myenteric plexus, and epithelial cells of mouse distal colon [11,19], and in the smooth muscle of the rat stomach and colon [20]. More recent immunohistochemical analysis confirmed the expression of the TRPM8 protein in the mouse colonic nerve endings distributed in the mucosal, submucosal, muscular, and serosal layers [21]. To date, human colonic TRPM8 expression appears to be related to pathological conditions. Transcripts encoding TRPM8 were detected in colon cancer but not in the corresponding normal human tissues [10] and increased TRPM8 expression has been revealed in colonic biopsy material from inflammatory bowel disease (IBD) patients as compared to healthy controls [11]. Secondly, because the chemosensory role is TRPM8’s most probable function in the digestive system, a TRPM8-dependent modulation of gastrointestinal motility is likely. TRPM8 activation seems to be involved in the menthol-induced relaxation of mouse [18] and human colon [22], in the cooling-induced contraction of rat gastric fundus [20], and guinea-pig ileum [23].

Recently, new chemical compounds able to modulate TRPM8 channels have been identified and they could be useful for understanding the implications of TRPM8 in pathophysiological processes [24]. In particular, a class of specific TRPM8 agonists belonging to the 1-[Dialkyl-phosphinoyl]-alkane (DAPA) has been reported to have a potential against pharyngeal irritation and inflammation [25,26,27]. Therefore, DAPA derivatives could find application in IBS, for treating symptoms such as irritation, inflammation, and muscular spasms.

The aims of the present study were (a) to verify the expression of TRPM8 in human distal colon; (b) to analyze the effects of TRPM8 activation on the mechanical activity of human distal colon; and (c) to characterize the mechanism of action responsible for the effects observed. Specifically, we analyzed the responses of human colonic circular smooth muscle to 1-[Diisopropyl-phosphinoyl]-alkane (DIPA) family (a subclass of DAPA) and compared the obtained responses to DAPA.

## 2. Results

### 2.1. TRPM8 Gene and Protein Expression in Human Distal Colon

**Figure 1:**
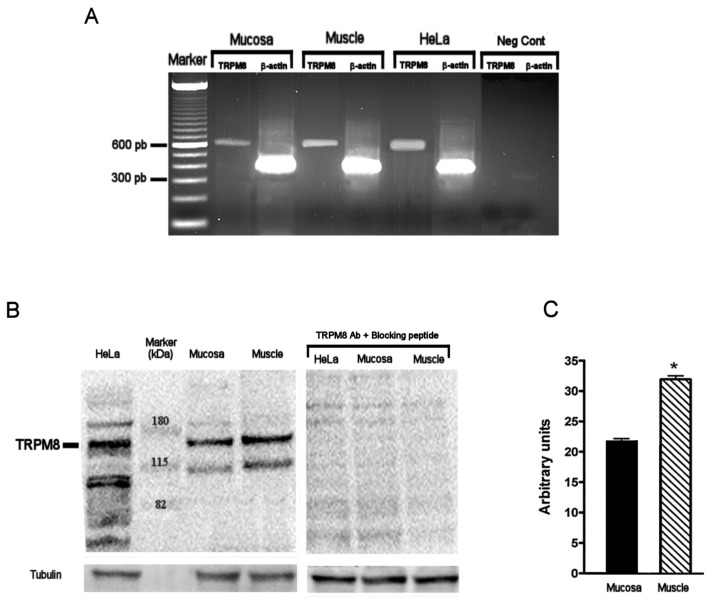
TRPM8 mRNA and protein expression in human distal colon. (**A**) Representative images of the reverse transcriptase-polymerase chain reaction (RT-PCR) results. A product of 621 bp corresponding to TRPM8 was detected in mucosa, smooth muscle, and HeLa cells used as positive control. The expression of β-actin (396 bp) was used as loading control. Negative control was obtained without addition of cDNA. (**B**) Western blot detecting protein levels for TRPM8 from colon mucosa and muscle. (**C**) Densitometric analysis of blots was performed using the NIH Image J 1.40 analysis program. HeLa cells were used as positive control. Human tubulin was used as loading control. Negative control was obtained by using blocking peptide added to TRPM8 Ab. *n* = 6; * *p* < 0.05 when compared to TRPM8 mucosa expression.

In the colonic specimens, reverse transcriptase-polymerase chain reaction (RT-PCR) analysis revealed the presence of a 621 bp product relative to TRPM8 mRNA expression, in both mucosa and smooth muscle layer (Figure 1A). Western blot analysis confirmed TRPM8 presence in distal colon, with higher expression in muscle than mucosa (Figure 1B,C). Indeed, two protein variants of TRPM8 of expected molecular weight of 140 and 112 KDa were detected. The faster running protein could represent one of the isoform variants of TRPM8 [28].

### 2.2. Functional Studies

**Figure 2:**
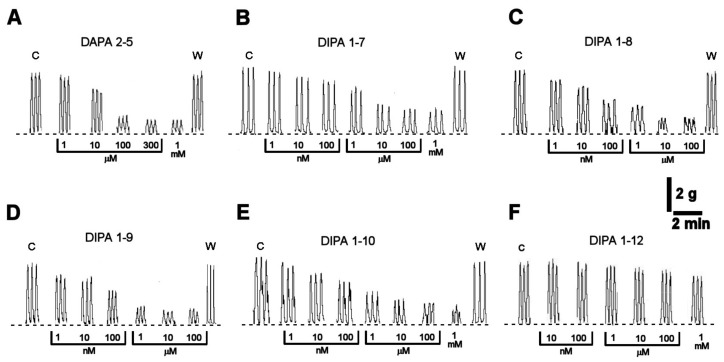
Typical recordings showing the inhibitory effects of increasing concentrations of DAPA 2–5 (1 μM–1 mM) (**A**), DIPA 1–7 (1 nM–1 mM) (**B**), DIPA 1–8 (1 nM–100 μM) (**C**), DIPA 1–9 (1 nM–100 μM) (**D**), DIPA 1–10 (1 nM–1 mM) (**E**), and DIPA 1–12 (10 nM–1 mM) (**F**) on the spontaneous contractions of human colon circular muscle. C = spontaneous contractions in control conditions. W = spontaneous contractions after washing out. Dotted line indicates the basal tone of the preparation.

**Figure 3:**
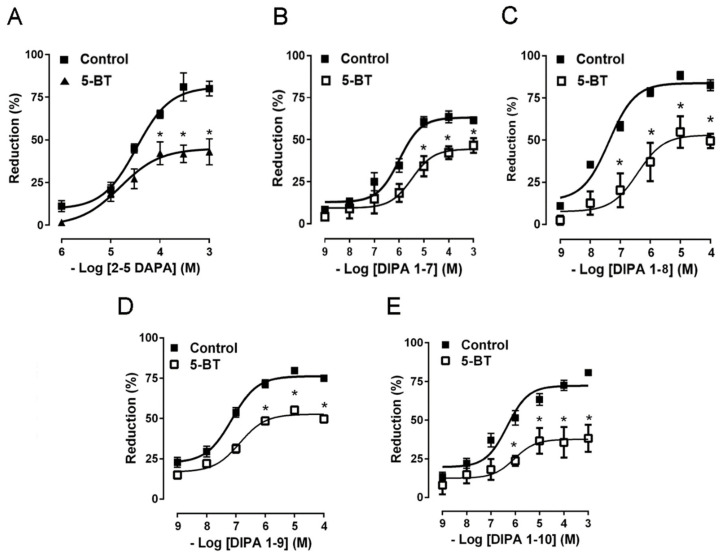
Concentration–response curves showing the inhibitory effects of increasing concentrations of DAPA 2–5 (1 μM–1 mM) (**A**), DIPA 1–7 (1 nM–1 mM) (**B**), DIPA 1–8 (1 nM–100 μM) (**C**), DIPA 1–9 (1 nM–100 μM) (**D**), and DIPA 1–10 (1 nM–1 mM) (**E**) on the spontaneous contractions of human colon circular muscle, in the presence or in the absence of 5-BT (1 μM). Data are means S.E.M. (*n* = 6 for each experimental conditions) and are expressed as percentage of inhibition of the spontaneous contractions. * *p* < 0.05 compared with the respective control conditions.

Circular muscle strips of human colon exhibited spontaneous mechanical activity consisting of phasic contractions at a frequency of 3 ± 0.3 contractions per minute and an amplitude of 4 ± 0.5 g (*n* = 36). The agonists DAPA 2–5 (1 μM–1 mM), DIPA 1–7 (1 nM–1 mM), DIPA 1–8 (1 nM–100 μM), DIPA 1–9 (1 nM–100 μM), and DIPA 1–10 (1 nM–1 mM) produced a concentration-dependent decrease in the amplitude of the spontaneous colonic contractions, without affecting the basal tone (Figure 2 and Figure 3). No agonist effect on frequency was observed (Supplementary Table S1). The inhibitory responses were reversible after washing out (Figure 2). DIPA 1–12 agonist (10 nM–1 mM) failed to significantly affect the colonic spontaneous contractions (Figure 2F).

The DIPA 1–8 agonist was the most efficacious and potent among the tested molecules, with EC_50_ = 41 nM Cls 28–61 nM and E_max_ = 88.3 ± 2.2 % (Table 1). In order to verify whether TRPM8 activation can induce relaxation, we tested the response to DIPA 1–8 (1 µM) of pre-contracted colon strips with carbachol (0.1 µM). As shown in Supplementary Figure S1, the TRPM8 agonist induced a rapid relaxation.

To assess the specificity of the effects, the preparations were pre-treated for 30 min with 5-BT (1 μM), a TRPM8 receptor antagonist. In presence of 5-BT, which per se did not affect the spontaneous mechanical activity, the inhibitory actions of TRPM8 agonists were significantly reduced (EC_50_ = 3.3 μM, Cls = 1.5–7 μM; EC_50_ = 374 nM, Cls = 181–772 nM; EC_50_ = 135 nM, Cls = 76–238 nM; EC_50_ = 911 nM, Cls = 206 nM–4 μM) for DIPA 1–7, DIPA 1–8, DIPA 1–9, and DIPA 1–10 respectively (Figure 3).

**Figure 4:**
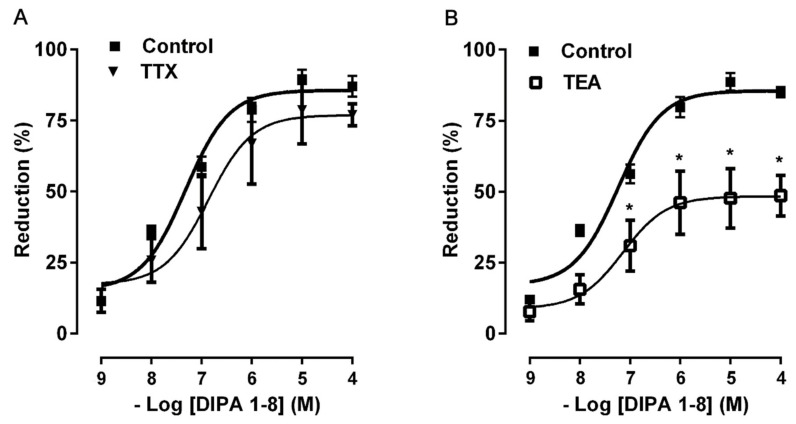
Concentration–response curves for the inhibitory effects induced by DIPA 1–8 (1 nM–100 μM) before and after TTX (1 μM) (**A**) and TEA (10 mM) (**B**). All values are means ± S.E.M (*n* = 6) and are expressed as percentage of inhibition of the spontaneous contractions. * *p* <0.05 compared with the respective control conditions.

In order to investigate the mechanism of action responsible for the inhibitory effect dependent on TRPM8 activation, the responses of DIPA 1–8 were tested in the presence of TTX (1 μM), a blocker of neural voltage-dependent Na^+^ channels. The pre-treatment of colonic samples with TTX, which per se failed to affect spontaneous contractions, did not modify the inhibitory responses to DIPA 1–8 at all concentrations tested (Figure 4A). On the contrary, TEA (10 mM), a non-selective blocker of K^+^ channels, significantly reduced the DIPA 1–8 inhibitory effect (Figure 4B) indicating an involvement of K^+^ channels in the inhibitory response to the TRPM8 agonist.

**Figure 5:**
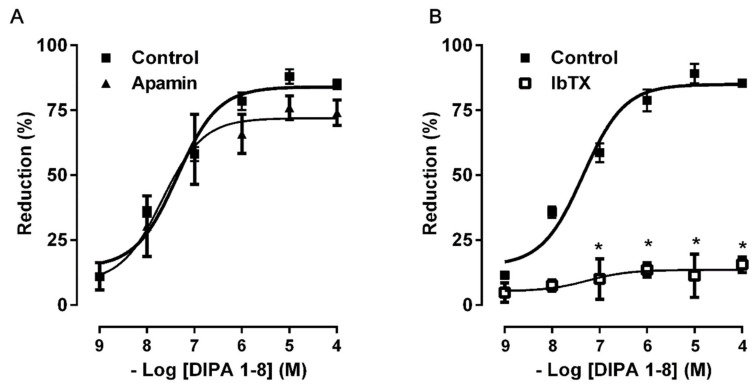
Concentration–response curves for the inhibitory effects induced by DIPA 1–8 (1 nM–100 μM) before and after apamin (100 nM) (**A**) and IbTX (10 µM) (**B**). All values are means ± S.E.M (*n* = 6) and are expressed as percentage of inhibition of the spontaneous contractions. * *p* <0.05 compared with the respective control conditions.

Moreover, the response to DIPA 1–8 was not affected by pre-treatment of colonic smooth muscle strips with apamin (100 nM), a blocker of small conductance Ca^2+^-dependent K^+^ channels (Figure 5A), while it was abolished by iberiotoxin (IbTX, 10 μM), a blocker of the large-conductance Ca^2+^-dependent K^+^-channels (Figure 5B).

## 3. Discussion

The results of the present study demonstrate that the TRPM8 receptors are expressed in the human distal colon and, once exogenously activated, are able to reduce the colonic smooth muscle contractility. The spasmolytic effects appear to be mediated by a direct action on the muscle cells, involving large-conductance Ca^2+^-dependent K^+^-channels.

The TRPM8 receptor is a non-selective cation channel, with a preference for Ca^2+^ permeation [5,29]. It shows multimodal gating being activated by cold (<28 °C), membrane depolarization, different cooling compounds such as menthol [29] and icilin, and changes in extracellular osmolality [5,29]. TRPM8 channels are highly expressed in peripheral sensory neurons (Aδ and C fiber afferents), and in deep visceral afferents in the prostate, bronchopulmonary tissue, bladder, and urogenital tract. TRPM8 channels are also expressed in the gut. In particular, TRPM8 gene and protein expression has been shown in the mucosal layer, muscle, and nerve endings of mouse and rat colon [11,20,21].

The current knowledge of TRPM8, mainly based on animal studies, concerns a role in thermosensation (primarily low temperatures) [30] and in visceral nociception [31]. In agreement, a recent study supports a pronociceptive role of TRPM8 in colitis-induced visceral hyperalgesia in mice [21]. However, evidence in mouse experimental colitis models has suggested protective effects of TRPM8 activation in colonic inflammation. Indeed, icilin treatment significantly attenuates induced colitis in wildtype mice, but not in TRPM8 deficient ones [4,11,21]. Therefore, whether TRPM8 modulation results in pro- or anti-nociception may depend on different conditions (noxious stimulus type, concentration of the pharmacological agonist) including the state of the TRPM8 receptor in healthy or inflamed tissue, which may explain contradictions between different studies.

While the involvement of TRPM8 channels in numerous human pathologies is well accepted [24], the importance of the TRPM8 channels in human physiology is less known. Indeed, in human intestine, TRPM8 expression has been associated with pathological conditions such as IBD [11] and colon cancer [10]. Although cramping pain, distension, and constipation are typical symptoms of IBD and IBS, no data on the TRPM8 role on gastrointestinal motility modulation are available. Indeed, recently human TRPM8 polymorphisms have been demonstrated to be associated with slower colonic transit [12].

Our study provides evidence, for the first time, for the presence of TRPM8 channels in human macroscopically healthy distal colon. Western-blot analysis showed a higher expression of TRPM8 channels in smooth muscle compared to the mucosa layer, suggesting that TRPM8 could have a potential role in the modulation of colon motor function. In fact, in our experimental conditions, the activation of the channels by exogenous specific synthetic TRPM8 agonists is able to reduce human circular smooth muscle spontaneous contractions, as well as to induce smooth muscle relaxation in CCh pre-contracted circular muscle strips. These results confirm the ability of peppermint oil (which contains the TRPM8 activator menthol as its biologically active component) to exert spasmolytic effects and inhibition of GI contractility [22,32,33].

The TRPM8 channels have attracted increasing attention in the past decade as promising drug targets for treatment of different pathologic processes, such as colonic inflammation [4,11,12,21], dry eye disease (DED) [26], tumors [34], oropharyngeal dysphagia [13], chronic cough [14], and hypertension [15]. Accordingly, numerous academic research groups and pharmaceutical companies have become interested in the pharmacological modulation of these receptors producing either agonists, antagonists, or both [35,36,37,38,39]. Among these, a recently synthetized class of TRPM8 agonists is represented by the 1-[Dialkyl-phosphinoyl]-alkane (DAPA) compounds [40]. DAPA are trialkyl derivatives of phosphoric acid, in which two of the alkyls are either sec-butyl (DAPA) or isopropyl (DIPA), and the third alkyl is C4 to C10. Previous work showed DAPA to be useful to treat skin and ocular discomfort [26] and heat edema as an anti-inflammatory agent [27,41].

In our experiments, we analyzed and compared the mechanical responses of the human distal colon to six DAPA analogs represented by DAPA 2–5, DIPA 1–7, DIPA 1–8, DIPA 1–9, DIPA 1–10, and DIPA 1–12. All tested substances, except DIPA 1–12, induced a concentration-dependent reduction of the spontaneous contraction amplitude of the circular smooth muscle. The DAPA-induced inhibitory effects were mediated specifically by TRPM8 channels, because they were significantly reduced by 5-BT, a TRPM8 receptor antagonist [42] at a concentration efficacious in reducing colonic inhibitory responses to WS-12 [22]. Different potencies of the DAPA analogues were found. The order of drug potency was DIPA 1–8 > DIPA 1–9 > DIPA 1–10 > DIPA 1–7 > DAPA 2–5. The different potency of DIPA compounds could depend on the number of carbon atoms present in the third alkyl, referred to as the 7-8-9-10-12 n-alkyl side-chain and corresponding to heptyl (DIPA 1–7), octyl (DIPA 1–8), nonyl (DIPA 1–9), decyl (DIPA 1–10), and dodecyl groups (DIPA 1–12), respectively. A high number of carbon atoms could be responsible for high complexity of the agonist molecular structure, reducing its ability to bind to the TRPM8 receptor. On the contrary, the decreased number of carbons could reduce the steric hindrance making the DAPA agonists more accessible to the TRPM8 receptor. In the same way, the presence of the n-butyl group in the second position (DAPA 2–5) might lead to a lesser ability of this agonist to link to the receptor. Our results are in accordance with previous data showing that the diisopropyl analogues (DIPA) were more active than the di-sec-butyl analogues (DAPA), and among DIPA, the nonane and octyl group permitted a longer duration of action than the decyl equivalent [26].

Another step of our research was to investigate whether TRPM8 activation reduces colon contractions via a direct action on the smooth muscle cells and/or via an indirect action involving neural pathways. To this aim, we chose DIPA 1–8 because it was more potent and efficacious than other agonists. TTX, a blocker of neuronal voltage-dependent Na^+^ channels, that per se failed to affect spontaneous contraction, indicating its balanced effect on excitatory and inhibitory nerves, did not modify the inhibitory effects of DIPA 1–8, suggesting that the response probably does not depend on TTX-sensitive neuronal activity, but is probably direct on the smooth muscle cells.

It is well known that K^+^ channels are involved in the control of the contraction of the gastrointestinal smooth muscle by setting resting potential and influencing slow waves and action potential configuration [43]. Activation of K^+^ channels causes membrane hyperpolarization of smooth muscle cells and therefore inhibition of Ca^2+^ influx through voltage-dependent L-type Ca^2+^ channels. In our experimental preparation, TEA, a non-selective K^+^ channel blocker, significantly antagonized the inhibitory response of DIPA 1–8, suggesting a potential role of the K^+^ channels in the myorelaxant action induced by the TRPM8 agonist. Because TRPM8 activation promotes Ca^2+^ entry into the cytosol by the TRPM8 Ca^2+^-permeable channels, the increased Ca^2+^ influx in the smooth muscle cell could be responsible for Ca^2+^-dependent K^+^ channel activation.

In our preparations, apamin, a blocker of the small conductance Ca^2+^-dependent K^+^ channels, did not affect the mechanical responses to DIPA 1–8, ruling out the involvement of these channels. On the contrary, IbTX, a blocker of the large-conductance Ca^2+^-dependent K^+^-channels, abolished the DIPA 1–8 effects, suggesting that these channels are involved in the reduction of colon contraction induced by TRPM8 activation. Consistent with our results, Silva et al. [44,45] observed that TRPM8 activation induced vasodilatation in rat mesenteric artery through an endothelium-independent pathway, which involved the activation of large-conductance calcium-activated potassium channels (BKCa) and inhibition of voltage-gated calcium channels. However, in rat internal pudendal artery, the relaxation induced by TRPM8 did not involve BKCa activation [46]. Therefore, it is not possible to generalize on the molecular mechanisms and further experiments by patch clamp are necessary to definitively demonstrate the role of the iberiotoxin-sensitive K^+^ channel in TRPM8-induced smooth muscle relaxation.

## 4. Materials and Methods

### 4.1. Human Tissue Specimens

The study protocol was approved by the Institutional Ethics Committee (HCP0617-June 2017; Comitato Etico CE Palermo 2 -ex D.A. n. 1360 del 16/07/2013) of the Azienda di Rilievo Nazionale ad Alta Specializzazione (A.R.N.A.S.), Ospedali Civico Di Cristina Benfratelli-Palermo. All patients provided written informed consent before inclusion in the study. Samples of human distal colon were collected from 36 subjects with no symptoms of major clinical motility disorders (aged 55–86, 32 males) who underwent colectomy for sigmoid cancer. Colonic samples were collected from macroscopically normal regions without any evidence of cancer lesions and placed in cold pre-oxygenated (95% O_2_ and 5% CO_2_) Krebs solution. Then, the mucosal layer was removed, and the specimens were stored overnight at 4 °C. Six samples (from 4 men and 2 women, aged 53–79) were used for biomolecular analysis. After phosphate-buffered saline, the scraped mucosa and the remaining tissue were separately collected in sterile tubes and stored at −80 °C.

### 4.2. TRPM8 Expression Analysis

Total RNA from the mucosa and smooth muscle was extracted using a PureLink RNA Mini Kit (Invitrogen, Carlsbad, CA, USA) according to manufacturer’s instructions and quantified by spectrophotometry. 1 mg of total RNA was reverse-transcribed using a High-Capacity c-DNA RT Kit (Applied Biosystems, Foster City, CA, USA). cDNA (5 µL; 30 ng total RNA equivalents per reaction) were denatured and subjected to RT-PCR amplification. The oligonucleotide primers were the following: For: 5′-cctgttcctctttgcggtgtggat-3′; Rev: 5′-tcctctgaggtgtcgttggcttt-3′ to generate a 621 bp product from human TRPM8. For: 5′-cgggatccccgccctaggcaccagggt-3′; Rev: 5′-ggaattcggctggggtgttgaaggtctcaaa-3′, to generate a 289 bp product from human β-actin. Each PCR cycle employed a 5 min denaturing step at 94 °C followed by 35 cycles at 95 °C for 15 s, 65 °C for 30 s, and 72 °C for 30 s, and a final extension step of 7 min at 72 °C. The amplimers were separated on a 1% agarose gel containing 0.5 mg/mL ethidium bromide for visualization. The gel was scanned under UV light. HeLa (Human epithelial carcinoma cell line) (purchased from ATCC, Manassas, VA, USA) was used as positive control [47].

For western blotting, the colon tissue (30 mg) was incubated on ice in RIPA buffer (50 mM Tris–HCl, pH 7.4; 150 mM NaCl, 1% Nonidet P-40) containing protease inhibitors (2 mM phenylmethylsulphonyl fluoride, NaVO_3_) for 1 h. Subsequently, it was centrifuged at 4 °C for 15 min at 13,000 g, and the supernatant was isolated. Protein concentration was measured by the Bio-Rad Protein Assay (Bio-Rad Laboratories, Hercules, CA, USA). Proteins (50 μg) were separated by 10% SDS-PAGE containing 0.1% SDS and transferred to Hybond-C nitrocellulose membranes (Amersham Life Science, Little Chalfont, UK) by electroblotting. Loading and transfer conditions were assessed by staining of the gel with Ponceau red. The relative migration position of the target protein was detected with a co-electrophoresed pre-stained molecular weight protein ladder (Invitrogen, Carlsbad, CA, USA). Subsequently the membranes were incubated overnight with antibodies to human TRPM8 (ab85617) (Abcam, Cambridge, UK; concentrated 1 μg/mL), with human tubulin (Abcam, Cambridge, UK; diluted 1:500) applied as a loading control. Regarding the specificity of TRPM8 immunoblotting, the primary TRPM8 antibody was pre-incubated for 2 h with the synthetic blocking peptide (ab95862) (Abcam, Cambridge, UK) before incubating with the membranes. Then the membranes were incubated with a goat anti-rabbit immunoglobulin G (IgG) secondary antibody conjugated to HRP (diluted 1:3000), recommended for TRPM8 detection (Santa Cruz Biotechnology, Dallas, TX, USA), or sheep anti-mouse IgG–HRP (diluted 1:10,000), recommended for tubulin detection (Amersham Pharmacia, Amersham, Buckinghamshire, UK). The target proteins were detected by enhanced chemiluminescence ECL (Pierce, Rockford, IL, USA). Once more, HeLa cell line was used as positive control (Anti-TRPM8 antibody-ab85617; https://www.abcam.com/trpm8-antibody-ab85617.html). Densitometric analysis of blots was performed using the NIH Image J 1.40 analysis program (National Institutes of Health, Bethesda, MD, USA).

### 4.3. Preparation of Circular Muscle Strips and Experimental Protocol

Methods used in the present study are the same as those previously reported [48]. Briefly, the circular muscle strips (4 mm wide by 10 mm long) were cut and suspended in a four-channel organ bath maintained at 37 ± 0.5 °C. Each chamber contained 8 mL of oxygenated Krebs solution with the following composition (mM): NaCl 119; KCl 4.5; MgSO_4_ 2.5; NaHCO_3_ 25; KH_2_PO_4_ 1.2; CaCl_2_ 2.5; glucose 11.1. One end of each strip was tied to organ holders, while the other end was secured with a silk thread to an isometric force transducer (FORT25, Ugo Basile, Biological Research Apparatus, Comerio, VA, Italy). The mechanical activity was digitized on an analog-to-digital converter, visualized, recorded, and analyzed on a personal computer using the PowerLab/400 system (Ugo Basile Biological Research Apparatus, Comerio, VA, Italy). A tension of 1 g was applied, and the tissues were allowed to equilibrate for 1 h. During this period, the strips developed spontaneous phasic contractions. In each experiment, up to six strips from the same specimen were studied. Preliminarily, in order to identify the most potent or efficacious TRPM8 agonist, we selected six selective TRPM8 receptor agonists (Table 2), to analyze the effects on colon mechanical activity. The agonists tested were trialkyl derivatives of phosphoric acid, in which two of the alkyls were either sec-butyl (DAPA) or isopropyl (DIPA) and the third alkyl was C4 to C10. Previously, the pharmacological actions on the TRPM8 channel have been validated in Chinese hamster ovary (CHO) cells transfected with human TRPM8 cDNAs [49] and tested in a mouse model of DED [26].

After the equilibration period, the effects induced by cumulative concentrations of DAPA 2–5 (1 μM–1 mM), DIPA 1–7 (1 nM–1 mM), DIPA 1–8 (1 nM–100 μM), DIPA 1–9 (1 nM–100 μM), DIPA 1–10 (1 nM–1 mM), and DIPA 1–12 (10 nM–1 mM) on the spontaneous mechanical activity were examined. The agonists were added to the bath, one by one, at increasing concentrations in volumes of 80 μL, and left in contact with the tissue for 4 min. The response to each TRPM8 agonist was tested in the presence of 5-benzyloxytryptamine (BT) (1 μM), a TRPM8 antagonist. In addition, the responses to DIPA 1–8, the most effective and potent agonist among those tested, were analyzed in the presence of tetrodotoxin (TTX; 1 µM), a voltage-dependent Na^+^-channel blocker, tetraethylammonium chloride (TEA), a non-selective blocker of K^+^-channels, apamin (100 nM), a blocker of small- conductance Ca^2+^-dependent potassium K^+^-channels, and iberiotoxin (IbTX, 10 µM), a blocker of the large-conductance Ca^2+^-dependent K^+^-channels. The concentrations of the blocker agents used were determined from the literature [22,50].

The effect of DIPA 1–8 (1 μM) was evaluated on the contractions evoked by carbachol (CCh, 0.1 μM). CCh (0.1 μM) induced reproducible and constant contractile responses, characterized by a fast initial peak, the phasic component, followed by a decline to a lower maintained tension level, the tonic component. CCh was left in contact with the tissue for 20 min and then washed out. DIPA 1–8 were added when the CCh contraction reached a plateau.

### 4.4. Drugs

The drugs used were the following: DAPA 2–5, DIPA 1–7, DIPA 1–8, DIPA 1–9, DIPA 1–10, and DIPA 1–12 (kindly supplied by Prof. Eddie Wei; Berkeley, CA, USA), TTX (Alomone Labs, Jerusalem, Israel), 5-BT hydrochloride, KCl, carbachol (CCh), tetraethylammonium chloride (TEA), apamin, and IbTX (Sigma-Aldrich, St. Louis, MO, USA). DIPA 1–8, DIPA 1–10, and DIPA 1–12 were dissolved in dimethylsulphoxide (DMSO) (0.1%). Control experiments using DMSO alone did not show any effects on the mechanical activity. All the other drugs were dissolved in distilled water. Chemicals were prepared as stock solution, which were diluted with Krebs solution on the experiment day.

### 4.5. Data and Statistical Analysis

The inhibitory effects of TRPM8 agonists were evaluated by measuring the mean amplitude of spontaneous contractions prior to and following drug administration. The results are expressed as the changes in mean amplitude of the phasic contractions and reported as percentages of the values obtained in the control (e.g., 100% corresponds to the abolition of spontaneous activity).

Concentration–response curves were computer-fitted to a sigmoidal curve using non-linear regression, and the concentration (EC50), with 95% confidence limits (Cls), producing half-maximum response, was calculated using Graph Pad Prism 6 Software (San Diego, CA, USA).

All data are expressed as mean values ± standard error of the mean (S.E.M.). The letter *n* indicates the number of human colonic samples. Statistical analysis was performed by means of Student’s *t*-test or 2-way ANOVA followed by Bonferroni post-hoc test, when appropriate. A probability value (*p*) of less than 0.05 was regarded as significant.

## 5. Conclusions

The results of the present study demonstrated that TRPM8 receptors are expressed in human distal colon and that ligand-dependent TRPM8 activation is able to reduce colonic spontaneous motility, probably by the opening of the large-conductance Ca^2+^-dependent K^+^-channels. The class of TRPM8 agonist belonging to dialkylphosphorylalkanes compounds, in particular the diisopropyl analogues (DIPA), represent promising drugs for the treatment of intestinal dysmotility. The effects of the DIPA 1–8 agonist on human colon emphasize the ability of TRPM8 channel activation to counteract IBS symptoms, such as pain, inflammation, and motility discomfort.

## Figures and Tables

**Table 1:** Potency and efficacy of the tested TRPM8 agonists (expressed as EC_50_ and E_max_ respectively) in determining reduction of human colon spontaneous contractions.

TRPM8 Agonist	Concentration Range	EC_50_	Cls	E_max_ (%)
DAPA 2–5	1 μM–1 mM	33 μM	21–50 μM	80.9 ± 8.2
DIPA 1–7	1 nM–1 mM	1 μM	0.5–2 μM	63.5 ± 3.5
DIPA 1–8	1 nM–100 μM	41 nM	28–61 nM	88.3 ± 2.2
DIPA 1–9	1 nM–100 μM	72 nM	42–123 nM	79 ± 1
DIPA 1–10	1 nM–1 mM	460 nM	251–977 nM	80.6 ± 1.8

EC_50_ = half-maximum response; Cls = 95% confidence limits; E_max_ (%) = maximum effect.

**Table 2:** TRPM8 agonists.

Code	Chemical Name	Chemical Structure
DAPA 2–5	1-Di(sec-butyl)phosphinoyl-pentane	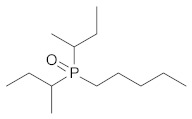
DIPA 1–7	1-Diisopropyl-phosphinoyl-heptane	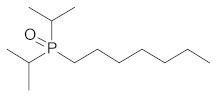
DIPA 1–8	1-Diisopropyl-phosphinoyl-octane	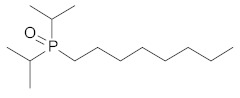
DIPA 1–9	1-Diisopropyl-phosphinoyl-nonane	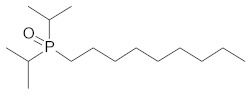
DIPA 1–10	1-Diisopropyl-phosphinoyl-decane	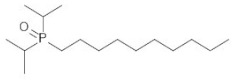
DIPA 1–12	1-Diisopropyl-phosphinoyl-dodecane	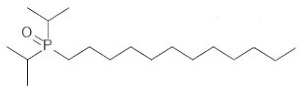

## Supplementary Materials

Supplementary materials can be found at https://www.mdpi.com/1422-0067/21/15/5403/s1, Table S1: Human colon spontaneous contraction frequency (cpm, contractions per minute) in control conditions or in the presence of the TRPM8 agonists; Figure S1: Typical tracings illustrating the response to DIPA 1–8 (1 µM) in the circular muscle strip of human colon precontracted by carbachol (CCh) (0.1 µM). W, washout. TRPM8 agonist induced a rapid relaxation.

## Abbreviations

5-BT	5-benzyloxytryptamine
ARNAS	Azienda di Rilievo Nazionale ad Alta Specializzazione-Highly Specialized National Relief Hospital
BKCa	Large-conductance calcium-activated potassium channels
CCh	Carbachol
CHO	Chinese hamster ovary cells
Cls	95% confidence limits
DAPA	1-[Dialkyl-phosphinoyl]-alkane
DED	Dry eye disease
DIPA	1-[Diisopropyl-phosphinoyl]-alkane
DMSO	Dimethylsulphoxide
EC_50_	Half-maximum response
E_max_	Maximum effect
HeLa	Human epithelial carcinoma cell line
HRP	Horseradish peroxidase
IBD	Inflammatory bowel disease
IBS	Irritable bowel syndrome
IbTX	Iberiotoxin
KCl	Potassium chloride
KDa	Kilodalton
RIPA	Radioimmunoprecipitation assay
RNA	Ribonucleic Acid
RT-PCR	Reverse transcriptase-polymerase chain reaction
SEM	Error of the mean
TEA	Tetraethylammonium chloride
TRP	Transient receptor potential
TRPM8	Transient receptor potential melastatin 8
TTX	Tetrodotoxin
