# Supplementary material for: TRPM8 Channel Activation Reduces the Spontaneous Contractions in Human Distal Colon
Source: Int J Mol Sci. 2020 Jul 29;21(15):5403. doi: 10.3390/ijms21155403 (PMC7432081; doi:10.3390/ijms21155403)
Supplement: Supplementary file 1 [file ijms-21-05403-s001.pdf]

Supplementary Materials

TRPM8 Channel Activation reduces the Spontaneous Contractions in Human Distal Colon

Table S1. Human colon spontaneous contraction frequency (cpm, contractions per minute) in control conditions or in the presence of the TRPM8 agonists.

	Control (cpm)	Agonist (cpm)	*p* Value
DAPA 2–5	4 ± 0.5	3.5 ± 1	0.4818
DIPA 1–7	2.9 ± 1.3	3.1 ± 1.2	0.5975
DIPA 1–8	3.5 ± 0.5	3 ± 0.5	0.2879
DIPA 1–9	3.2 ± 1.1	3 ± 1	0.8272
DIPA 1–10	3.5 ± 1	3.4 ± 0.9	0.9038

The DIPA agonist had no effects on frequency contractions. A probability value (p) of less than 0.05 was regarded as significant.

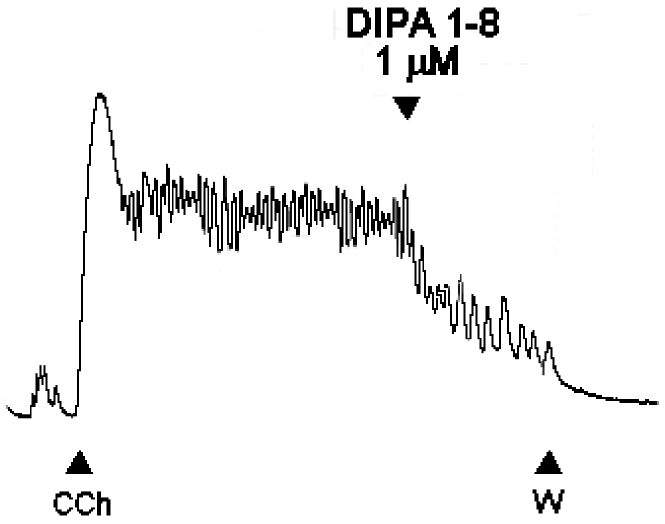

Figure S1. Typical tracings illustrating the response to DIPA 1–8 (1 μM) in the circular muscle strip of human colon precontracted by carbachol (CCh) (0.1 μM). W, washout. TRPM8 agonist induced a rapid relaxation.
